# Dynamics of Open DNA Sliding Clamps

**DOI:** 10.1371/journal.pone.0154899

**Published:** 2016-05-05

**Authors:** Aaron J. Oakley

**Affiliations:** School of Chemistry University of Wollongong, Wollongong 2522, NSW, Australia; Tulane University Health Sciences Center, UNITED STATES

## Abstract

A range of enzymes in DNA replication and repair bind to DNA-clamps: torus-shaped proteins that encircle double-stranded DNA and act as mobile tethers. Clamps from viruses (such as gp45 from the T4 bacteriophage) and eukaryotes (PCNAs) are homotrimers, each protomer containing two repeats of the DNA-clamp motif, while bacterial clamps (pol III β) are homodimers, each protomer containing three DNA-clamp motifs. Clamps need to be flexible enough to allow opening and loading onto primed DNA by clamp loader complexes. Equilibrium and steered molecular dynamics simulations have been used to study DNA-clamp conformation in open and closed forms. The *E*. *coli* and PCNA clamps appear to prefer closed, planar conformations. Remarkably, gp45 appears to prefer an open right-handed spiral conformation in solution, in agreement with previously reported biophysical data. The structural preferences of DNA clamps in solution have implications for understanding the duty cycle of clamp-loaders.

## Introduction

In all three domains of life (and some viruses), DNA polymerases are responsible for DNA synthesis during the replication, recombination, and/or repair of chromosomal DNA. A common feature of replicative and other DNA polymerases is their association with a “sliding clamp”. These DNA clamps act as mobile double-stranded (ds) DNA-encircling tethers for many proteins in DNA metabolism [1]. The earliest references to DNA clamps were as DNA polymerase processivity factors. For example, the *E*. *coli* clamp was called copolymerase III by the Kornberg laboratory [2]. The first of such processivity factors to be identified as a sliding clamp was gp45 from bacteriophage T4, essential for processive DNA replication by T4 DNA polymerase gp43 [3]. In eubacteria, the sliding clamp is the β-subunit of the replicative DNA polymerase III (Pol III) holoenzyme [4]. The first eukaryote sliding clamp was identified in human cells as the “proliferating cell nuclear antigen” (PCNA), so-named for its identity as an antigen in proliferating cells in the sera of patients with systemic lupus erythematosus [5]. Sliding clamps from the hyperthermophilic archaeon *Pyrococcus furiosus* and several other archaea are homologous to eukaryotic PCNA [6]. Sliding clamps interact with many proteins in DNA replication and repair. Direct interaction with the replicative DNA polymerases (e.g. Pol δ and Pol ε with PCNA in eukaryotes, Pol III α with Pol III β in eubacteria, gp43 with gp45 in T4 bacteriophage) enhance the processivity of their DNA synthesis.

**Fig 1:**
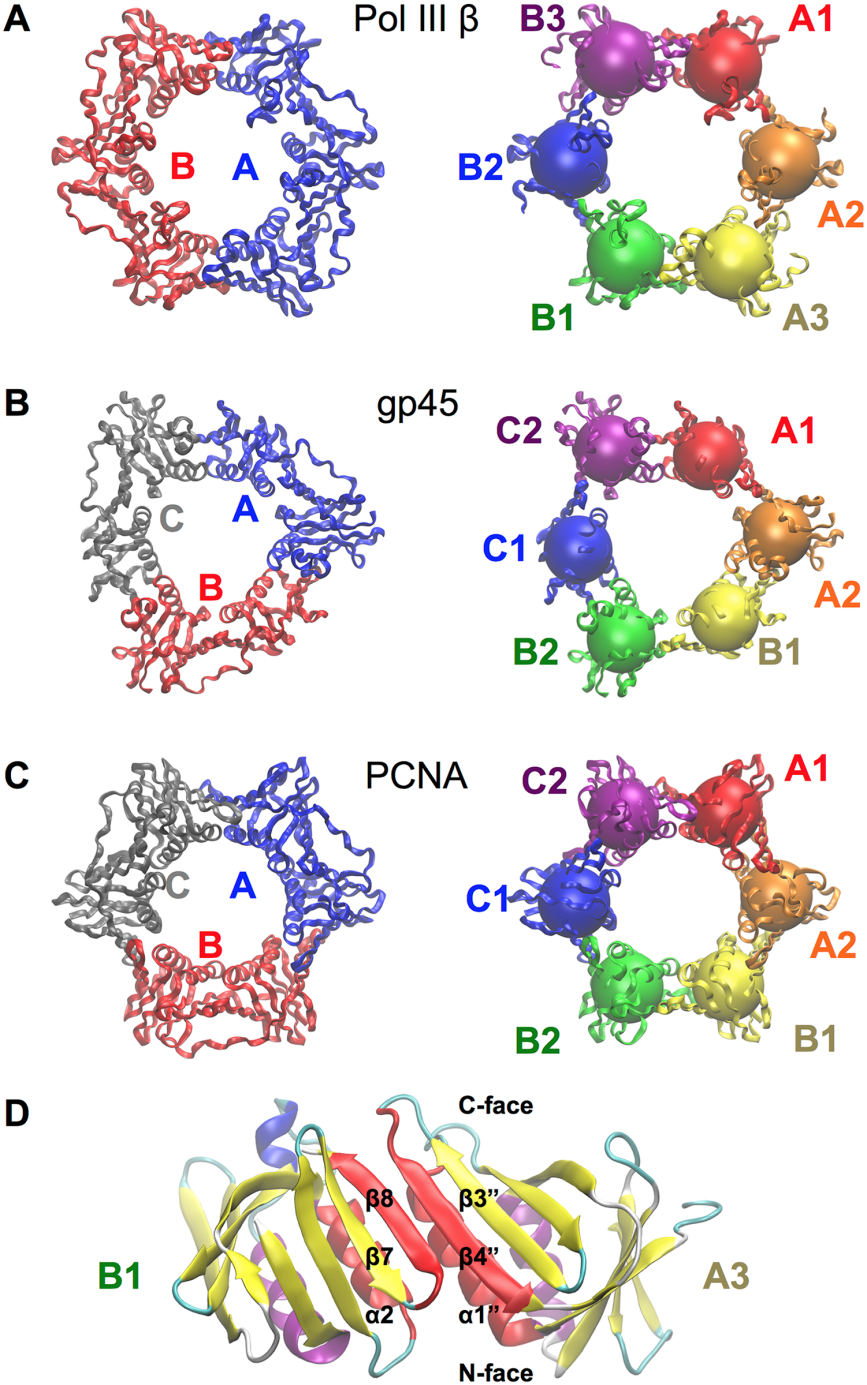
Structures of DNA clamps. Ribbon diagrams of (A) pol III β (PDB ID 1OK7) (B) gp45 homolog from bacteriophage RB59 (PDB ID 1B77) and (C) PCNA (PDB ID 1PLQ). In the left-hand column each protomer is labelled. In the right-hand column each clamp domain is drawn in a different colour and labelled. Spheres indicate the centre of mass of each clamp domain. Two DNA clamp domains of pol III β are shown in (D). The α-helix and β-strands forming the inter-domain interactions are highlighted in red.

DNA clamps form toroidal structures [7] (Fig 1A–1C). The first sliding clamp structure determined was *E*. *coli* β [8]. Representative crystal structures of human [9], yeast [10], and archaeal PCNA [6], phage T4 gp45 [11], and others have also been solved. Bacterial DNA clamps observed to date form head-to-tail homodimers, with each protomer containing three copies of the “DNA-clamp” motif. PCNA and phage homologs are composed of three monomers, each containing two copies of the “DNA-clamp” domain. Thus, all of these DNA clamps have pseudo six-fold symmetry. Individual DNA-clamp domains consist of two α-helices and two β-sheets. Interactions between adjacent domains involve packing of adjacent α-helices and the formation of a continuous curved β-sheet spanning across the domain boundaries (Fig 1D). Recently, PCNAs from *Sulfolobus tokodaii* (stoPCNA2 and stoPCNA3) were observed to form a heterotetramer proposed to function as a Holliday junction clamp [12]. The structures of bacterial [13] and eukaryotic [14] sliding clamps in complex with DNA have been determined; dsDNA was observed to pass through the central cavity of the clamps in both cases.

Essential to the function of sliding clamps are so-called clamp loaders that open and mount sliding clamps onto the 3’ ends of primer–template junctions. Clamp loaders across all domains of life and some bacteriophages are pentameric complexes of proteins containing AAA+ family ATPases [4, 15]. The *E*. *coli* clamp loader, composed of the Pol III τ/γ, δ and δ' subunits, is also known as the γ complex. It contains one copy each of the δ and δ' subunits and three in total of τ and/or γ subunits. The phage T4 clamp loader complex is composed of two proteins: gp44 and gp62 in a 4:1 ratio. In eukaryotes and archaea, the clamp loader is known as replication factor C (RFC). The *S*. *cerevisiae* RFC is composed of five different subunits named Rfc1, Rfc2, Rfc3, Rfc4 and Rfc5. Crystal structures of clamp loaders from *E*. *coli* [16–18], *S*. *cerevisiae* RFC [19] and phage T4 [20] have been determined. Each clamp loader subunit contains three domains: domains I and II form the AAA+ ATPase module, and the C-terminal domains III form a “collar” that oligomerizes the clamp loader. ATP binding to AAA+ domains triggers the formation of a spiral arrangement [17], which in turn allows binding to primer–template DNA. ATP binding also enables the clamp loader to bind and open the sliding clamp. The spiral formed by the clamp loader AAA+ domains and bound sliding clamp matches the symmetry of DNA, *i*.*e*. a right-handed helix [17, 20].

Several lines of evidence suggest that DNA-clamps exhibit structural fluctuations in solution. Hydrogen exchange mass spectrometry was used to characterize the conformation and dynamics of *E*. *coli* β and suggest that the interfaces between clamp monomers open transiently [21]. Backbone amide exchange, chemical and thermal denaturation experiments show that human PCNA is less stable in solution than the *S*. *cerevisiae* homolog, notwithstanding their near-identical structures [22, 23]. The gp45 trimer is less stable in solution than human PCNA trimers, which are in turn less stable than the *E*. *coli* β dimers. Furthermore, while PCNA and β dissociate slowly from circular DNA (t_1/2_ ≈ 24 and ≈ 60 min, respectively) [24] gp45 requires polymerase binding to stabilize it on DNA [25]. Analytical ultracentrifugation and fluorescence data suggest that one of the gp45 subunit interfaces is open in solution [26]. Time-resolved Förster resonance energy transfer (trFRET) of gp45 with fluorescent labels at the subunit interface gave a bimodal tfFRET distance distribution, indicating two closed interfaces and one open interface [27]. Using fluorescence correlation spectroscopy, *E*. *coli* Pol III β was shown to be stable in solution as a closed ring at concentrations three orders of magnitude lower than *S*. *cerevisiae* PCNA [28].

There has been increasing interest in the use of molecular dynamics (MD) simulations to understand the dynamical behavior of DNA clamps. Dynamics simulations of PCNA from *S*. *cerevisiae*, *H*. *sapiens*, and *Pyrococcus furiosus* from which one of the three subunits was removed showed structural fluctuations corresponding to lateral and out-of-plane distortions of the clamp resulting from bending and twisting of the β-sheets [29, 30]. Simulations of human and *Archaeoglobus fulgidus* PCNA trimers surrounding dsDNA predicted a tilted orientation of clamps with respect to the main axis of DNA that optimizes interactions between the phosphodiester backbone and basic residues lining the PCNA inner surface [31]. These predictions agreed well with the subsequent crystal structure of *S*. *cerevisiae* PCNA with DNA [14]. Steered MD simulations on *S*. *cerevisiae* PCNA and the *E*. *coli* Pol III β were used to show distinct opening mechanisms (unzipping in PCNA; abrupt cooperative disruption in *E*. *coli* Pol III β) [32]. Similarly, steered MD simulations suggest that human PCNA opens by unzipping of the dimer interface [33].

In this study, MD simulations were used to probe the structures of three distinct types of DNA clamp (bacteriophage, eukaryote and bacterial) in solution, in different oligomerization states. Steering forces were used to produce open DNA-clamps in different conformations, and long equilibration runs were used to observe their subsequent relaxation. To our knowledge, this is the first study in which bacteriophage, eukaryote and bacterial clamps were subject to identical simulation schemes and in different oligomerization states, and affords comparison of their behavior.

## Methods

For simulations, representatives of eukaryotic, bacteriophage and bacterial clamps were selected from the PDB based on resolution and structure quality. These were *S*. *cerevisiae* PCNA (PDB ID 1PLQ) [10], the gp45 homolog from bacteriophage RB59 (PDB ID 1B77) [34] and *E*. *coli* Pol III β (PDB ID 1OK7) [35]. (The gp45 homologs from RB59 and T4 are very similar: sequence identity 79%, similarity 87%) All MD trajectories were calculated by NAMD [36] using the CHARMM27 all-atom force field [37, 38]. Structures were embedded in cubic water boxes. Sodium and chloride ions were added (target salt concentration 100 mM) such that the systems had zero net charge. All simulations were run as NpT ensembles (temperature 310 K; pressure 101.325 kPa) with periodic boundary conditions. Temperature was controlled using Langevin dynamics (damping constant 5 ps^–1^). Pressure control used the Nosé-Hoover Langevin piston (period 100 fs; decay rate 50 fs). A multiple time-step approach was used with 1, 2, and 4 fs for bonded, non-bonded and long-range electrostatic calculations respectively. The Particle-mesh Ewald with a grid resolution of ≤ 1 Å was used to calculate long-range electrostatic forces. van der Waals’ interactions were smoothly scaled to zero between 10 and 12 Å. All systems in this study were subjected to energy minimization (10,000 steps) prior to equilibration by MD. Intact gp45, PCNA and Pol III β clamps were equilibrated for 50 ns and were used as starting points for steered MD simulations. Monomers of gp45, PCNA and β, and dimers of gp45 and PCNA were equilibrated for 100 ns.

Three types of open clamp models (designated 1, 2 and 3) were produced for gp45, PCNA and β-clamps using the collective variables capabilities of NAMD [39]. For model type 1, the distances between centers-of-mass of residues at a monomer–monomer interface (one α-helix and β-strand equivalent to those indicated in Fig 1D) were steered apart at constant velocity *v* = 2.0 Å ns^–1^ using a simple harmonic potential. Test calculations showed that a tight harmonic potential (force constant 500 kcal mol^–1^ Å^–2^) applied to the distance between the centers-of-mass of the two groups (about 10 Å at the start of each simulation) resulted in smooth opening of the interface. The distances were increased by 20 Å from their initial values in all cases. For models of type 2, the clamps in open left-handed spiral conformations were produced, and for type 3 models, open right-handed spiral conformations were produced. Three torsion angles defined by the centers-of-mass of DNA-clamp domains around the interface were steered. In the case of Pol III β, the torsion angles were defined by domains B1-B2-B3-A1, B2-B3-A1-A2, and B3-A1-A2-A3 (opening the A3/B1 interface). For gp45 and PCNA, the torsion angles were defined by domains B1-B2-C1-C2, B2-C1-C2-A1 and C1-C2-A1-A2 (opening the A2/B1 interface). Test calculations showed that a tight harmonic potential (50 kcal mol^–1^ °^–2^) opened the clamps smoothly when applied to the torsion angles. For type 2 models, a decrease of 20° in each of the three dihedral angles induced the formation of a left-handed spiral. For type 3 models, the same torsion angles were increased by 20°. In all cases, steering forces were applied for 10 ns, after which the systems were allowed to equilibrate for a further 140 ns without any restraining forces.

All trajectory data were analyzed in VMD [40]. Relative movements in clamp domains were analyzed by measuring distances, angles and dihedral angles between the centers of mass of the clamp domains (as shown in Fig 1). Where equilibrium values for geometric parameters are reported, averages and standard deviations are given, discarding the first 10 ns as pre-equilibration. To compare the fluctuations of assemblies of different sizes the normalized RMSD [41], RMSD_100_ was used Eq (1),

$$RMSD_{100}=\frac{RMSD}{1+\text{ln}\left(\sqrt{\frac{N}{100}}\right)}$$ (1)

where N is the number of residues.

To better visualize the changes to the DNA-clamps, a coarse-grained representation method was developed whereby each of the clamp domains was represented as a sphere. A vector connecting each sphere to the center-of-mass of the system is calculated and represented as an arrow. Throughout the simulation, the length and direction of each vector is kept constant with respect to the position and orientation of the individual domains. Thus, changes in the orientation of each clamp domain are visualized as changes in the direction of the arrow.

## Results

**Fig 2:**
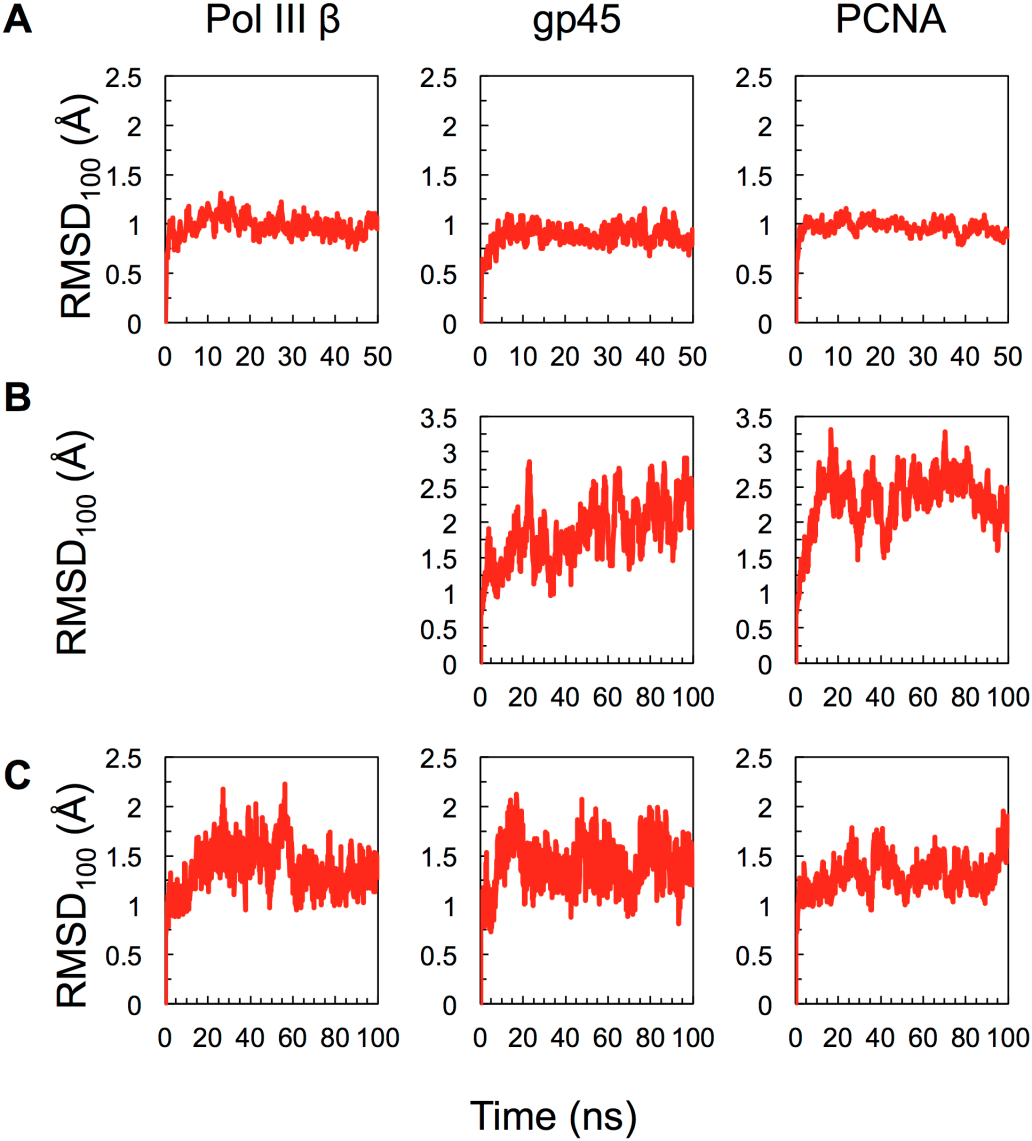
RMSD_100_ as a function of time in MD simulations. (A) intact pol III β, gp45 and PCNA clamps, (B) gp45 and PCNA dimers and (C) pol III β, gp45 and PCNA monomers.

The various systems simulated are shown in Table 1. The intact DNA clamps change little from their initial structure over 50 ns as demonstrated by RMSD_100_ values rising to only about 1 Å (Fig 2).

**Table 1:** Summary of MD simulations.

System	protomers	Total number of atoms
Pol III β monomer	1	94,811
Gp45 monomer	1	55,390
PCNA monomer	1	55,482
Gp45 dimer	2	124,385
PCNA dimer	2	125,493
Pol III β dimer	2	125,625
Gp45 trimer	3	125,169
PCNA trimer	3	125,470

### Simulations of Pol III β

**Fig 3:**
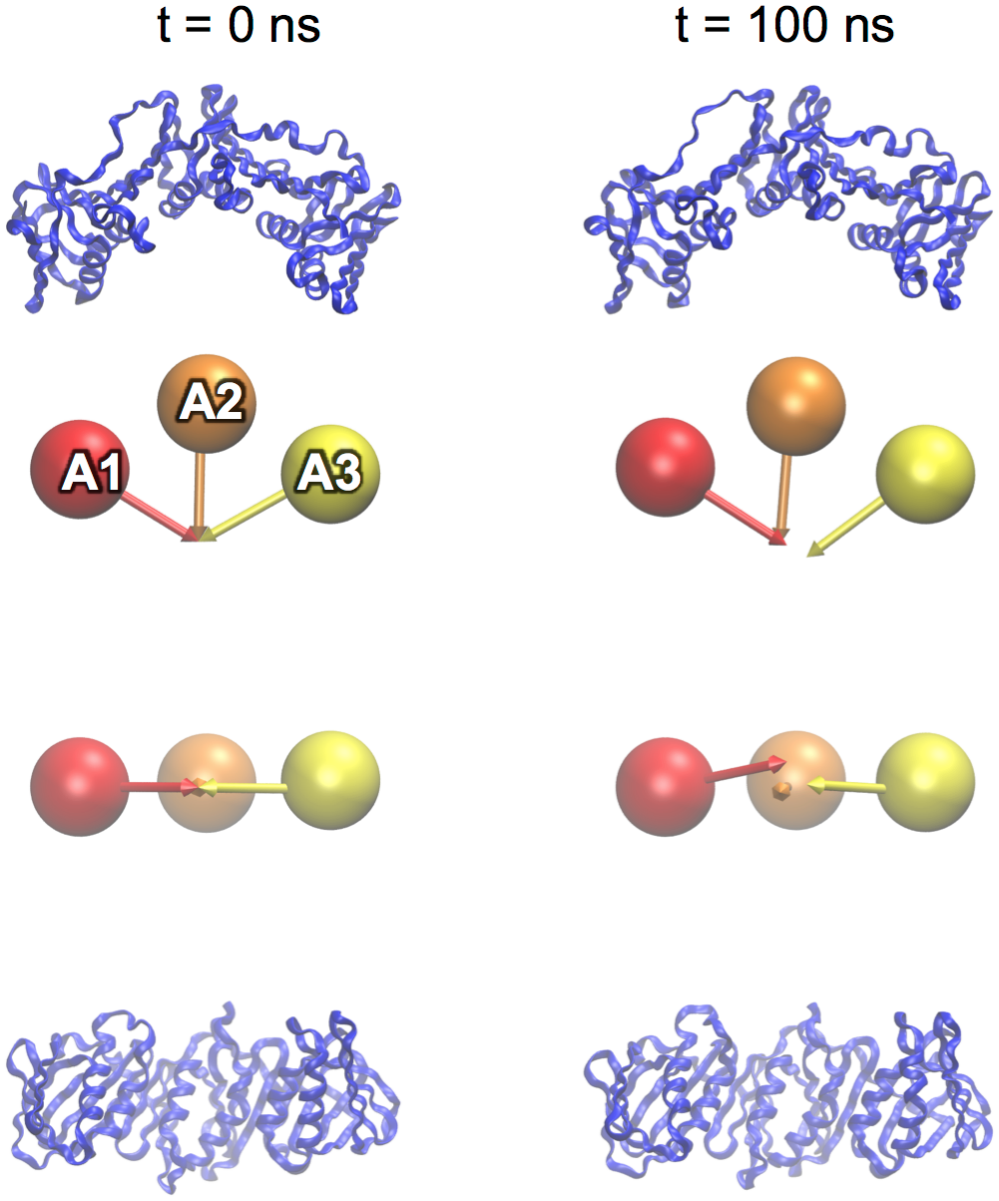
The Pol III β monomer. Clamp domains are represented in ribbon form and as “spheres and arrows” as described in the methods. The β monomer is shown in its initial conformation (left; t = 0 ns) and after 100 ns of equilibration (right; t = 100 ns). Orthogonal views are shown.

The β dimer remains stable (RMSD_100_ ≈ 1 Å) over 50 ns and shows no substantial deviation from the crystal structure and by the same measure, the β monomer is more flexible in isolation (Fig 2A and 2C). Simulations of the monomer show that inter-domain distances and angles remain close to initial values (i.e. the conformation observed in the dimer crystal structure), domain A1 rotates with respect to domain A2 as would occur during the formation of an open, right-handed spiral. Domain A3 rotates away from domain A2 in an “in-plane” motion, increasing the A1-A2-A3 angle from 123.1° in the starting conformation to 130.0 ± 2.8° (Fig 3).

### Simulations of gp45

**Fig 4:**
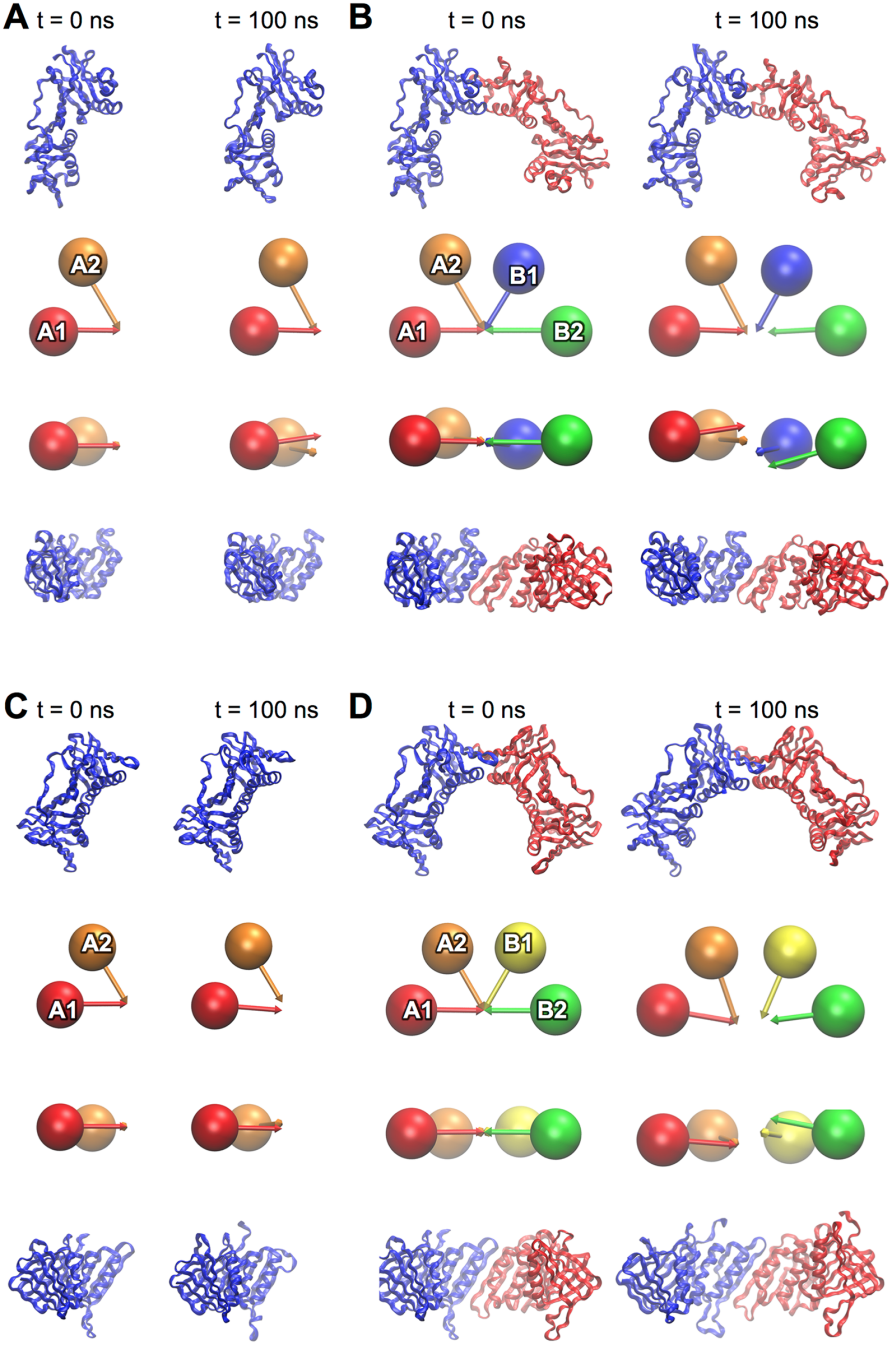
gp45 and PCNA monomers and dimers. A gp45 monomer (A) and (B) dimer are represented in ribbon form and as “spheres and arrows” as described in methods. A PCNA monomer (C) and dimer (D) are represented in an identical fashion. In all cases the starting structures for the simulations are shown at left (t = 0 ns), and the same structures after equilibration (t = 100 ns) are shown at right. In all cases orthogonal views are shown.

While the closed gp45 trimer deviates little with respect to the crystal structure during equilibration (RMSD_100_ ≈ 1 Å; Fig 2A), the gp45 dimer (Fig 2B) and monomer (Fig 2C) drift away from their initial conformations. Both dimer and monomer form arrangements whereby each domain is rotated slightly with respect to its neighbor (Fig 4A and 4B) consistent with the adoption of a right-handed spiral. The clamp domains in the gp45 monomer rotate 13.9 ± 4.4° with respect to each other, and similar rotations between domains are seen in the gp45-dimer (Fig 2B). Rotations were observed between domains A1 and A2 (11.8 ± 5.2°), B1 and B2 (8.3 ± 4.2°), while rotations between domains A2 and B1 were less pronounced (4.1 ± 5.9°). The net effect of these rotations is to generate a right-handed spiral conformation. These movements do not appear to be driven by localized changes in the structure but are due to an overall relaxation of the structures.

### Simulations of PCNA

Like the Pol III β dimer and gp45 trimer, the PCNA trimer remains stable and deviates little with respect to the initial structure (RMSD_100_ ≈ 1 Å) (Fig 2A). In monomeric form, the DNA-clamp domains of PCNA are more flexible (Fig 2C), but do not deviate substantially from their initial conformation (Fig 4C). The most pronounced changes occur in the simulation of the PCNA dimer: the RMSD_100_ fluctuates between 1.5 and 3 Å. PCNA dimers appear to undergo hinging motions about the A2-B1 interface (Fig 4D). This corresponds to an increase in the A1-A2-B1 and A2-B1-B2 inter-domain angles from 120.5° to 129.1 ± 2.3° and from 119.3 to 126.5 ± 2.5°, respectively. Small rotations were observed between domains such that a slight right-handed helical conformation was apparent: A1 and A2 (–5.9 ± 4.2°), A2 and B1 (–8.5 ± 4.6) B1 and B2 (–5.5 ± 3.3°).

### Clamp opening by steered MD simulations

**Fig 5:**
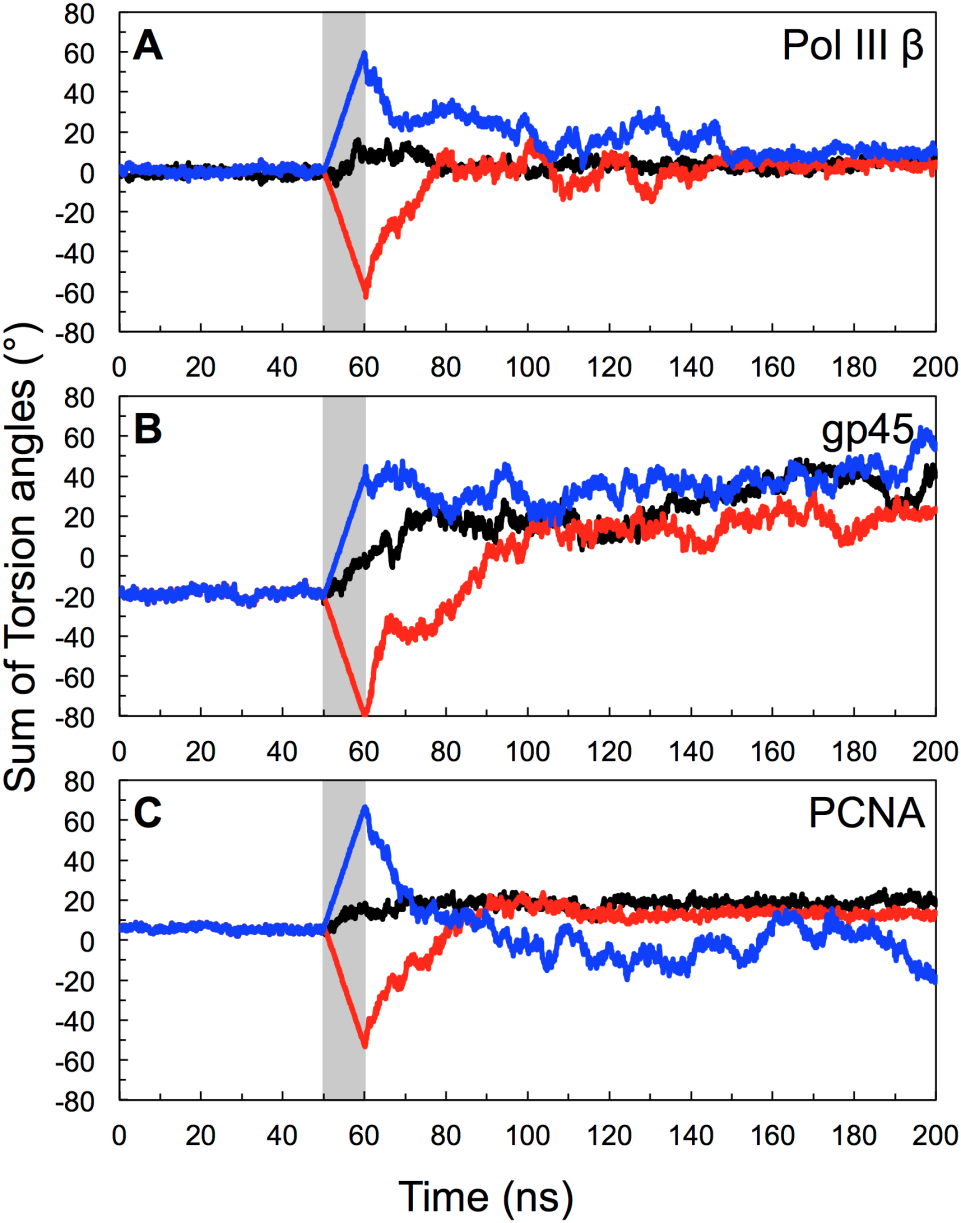
Sum of the internal torsion angles around the interface being broken during MD simulations. (A) pol III β, (B) gp45 and (C) PCNA. Positive and negative values indicate right-handed and left-handed spirals, respectively. The period when steering forces were applied (from 50 to 60 ns) is shaded grey. The traces are coloured according to model type: 1 (open interface; black), 2 (left-handed spiral; red) or 3 (right-handed spiral; blue).

**Fig 6:**
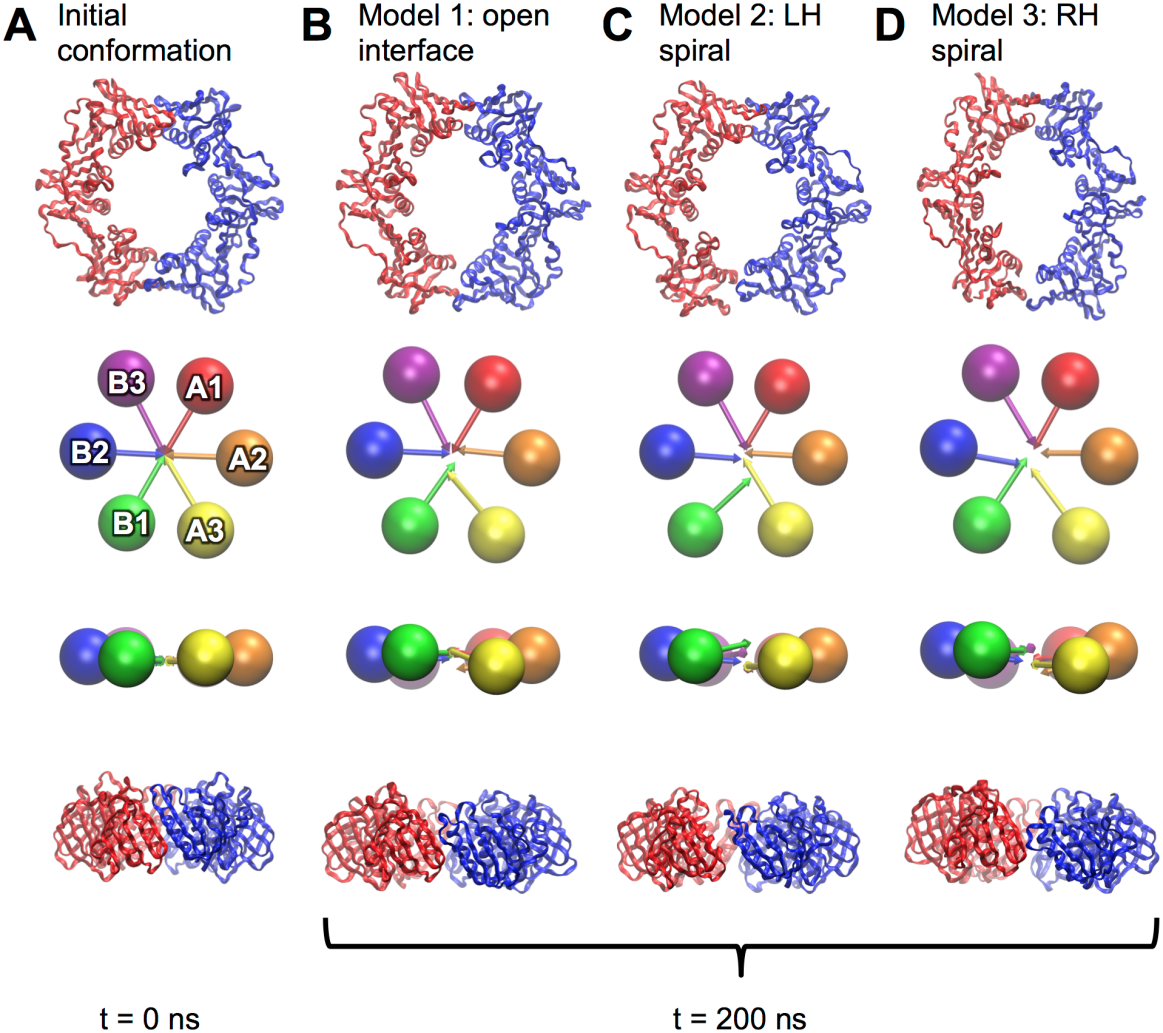
Structures of pol III β before and after ring-opening simulations. Initial conformation shown in (A) and at 200 ns of equilibration for models of type 1 (B), type 2 (C) and type 3 (D). All clamp domains are represented as ribbons and as “spheres and arrows” as described in methods. Pairs of orthogonal views are shown in all cases.

**Fig 7:**
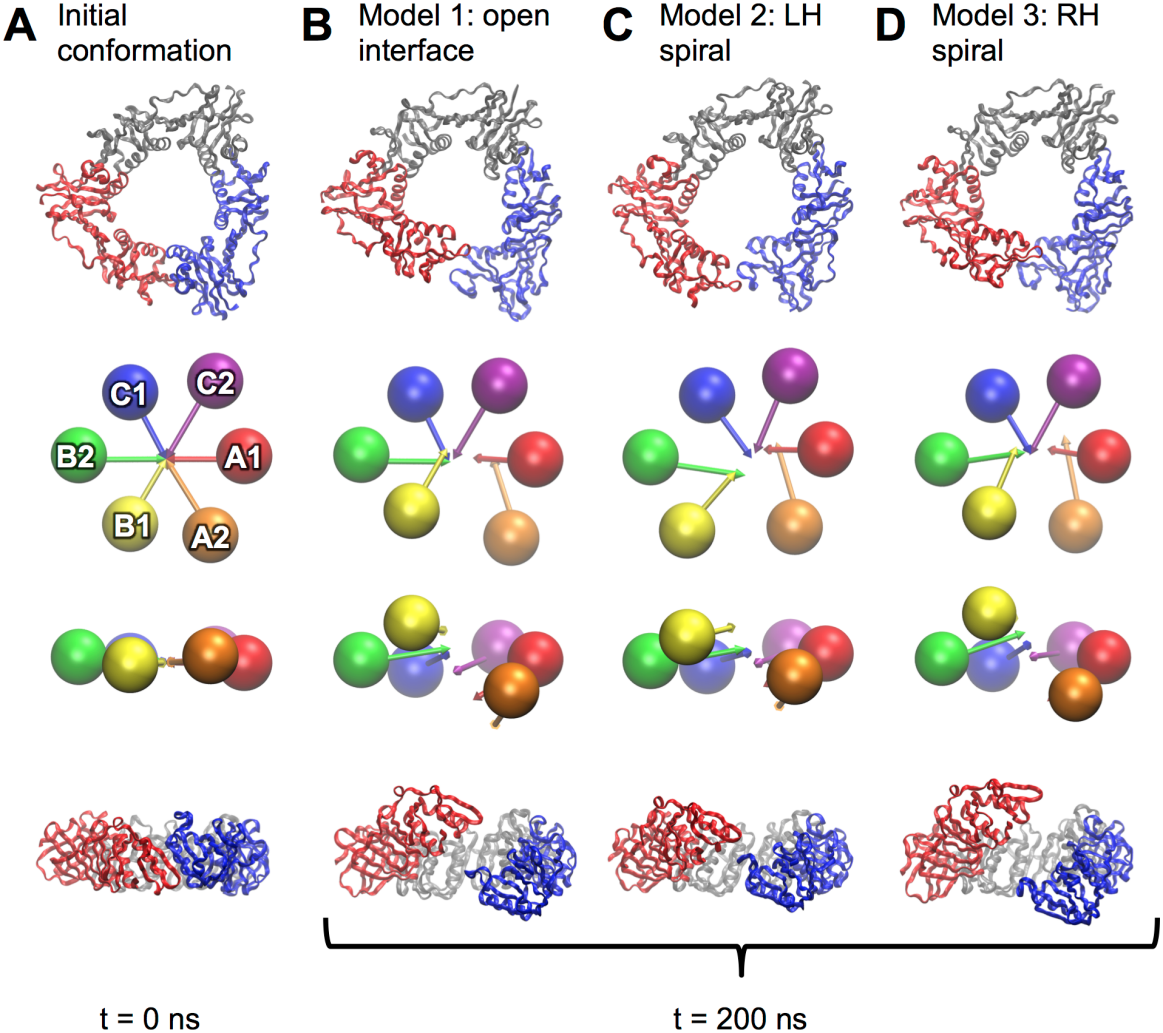
Structures of gp45 before and after ring-opening simulations. Initial conformation shown in (A) or after 200 ns of equilibration for models of type 1 (B), 2 (C) and 3 (D). All clamp domains are represented as ribbons and as “spheres and arrows” as described in methods. Pairs of orthogonal views are shown in all cases.

Steering forces were successfully applied to DNA clamps to create models of open clamp conformations. Intriguingly, in all simulations of type 3 models (opening the interface in the direction of a right-handed spiral), the physical mechanisms were the same: for β, gp45 and PCNA the beta-sheets at the domain interface slide past each other in the direction of the beta strands. Opened β models showed a tendency to close with further equilibration (Figs 5A and 6A). Strikingly, the gp45 clamp remained open, and adopted right-handed spiral configurations, regardless of the method by which they were opened (Figs 5B and 7).

The *E*. *coli* β models showed a preference for closed structures. Following ring opening *via* any of the three steered MD regimes, the opened clamp interfaces re-associate when relaxing the steering forces (Fig 6). When the interface was steered apart (model 1), the clamp remained relatively planar. The beta strands of domains A3 (β4”) and B1 (β8) unzipped from the N-face upward, and there was a concerted breaking of interactions followed by opening of the interface. 10 ns after steering forces were removed, re-association of the interface occurred with the interface helix of domain A3 initially forming interactions with the N-terminal end of strand β8 in domain B1 followed by association of the hydrophobic side-chains of the interface, followed at the end by the formation of hydrogen bonds between strands β4” and β8. Further translation of the domains in the direction of these strands is required to re-from the native structure. When β is forced to adopt an open left-handed spiral (model 2), the interface breaks in a concerted manner. Upon equilibration, the ring rapidly flattens out, and the open interfaces of domains A3 and B1 re-associate. Opening of β to form a right-handed spiral (model 3) occurs smoothly, with the domains slipping past each other. When the open RH spiral starts to close, the spiral arrangement flattens, domains A3 and B1 move closer together and re-associate.

Simulations on the open gp45 clamp indicate a clear preference for an open conformation in solution. All opened clamp models, when allowed to equilibrate, resulted in a right-handed spiral configuration (Fig 7). When the interfaces were steered apart (model 1), or a right-handed spiral induced (model 3), they slid past each other. Only when open as a left-handed spiral did the interface open in a concerted manner.

**Fig 8:**
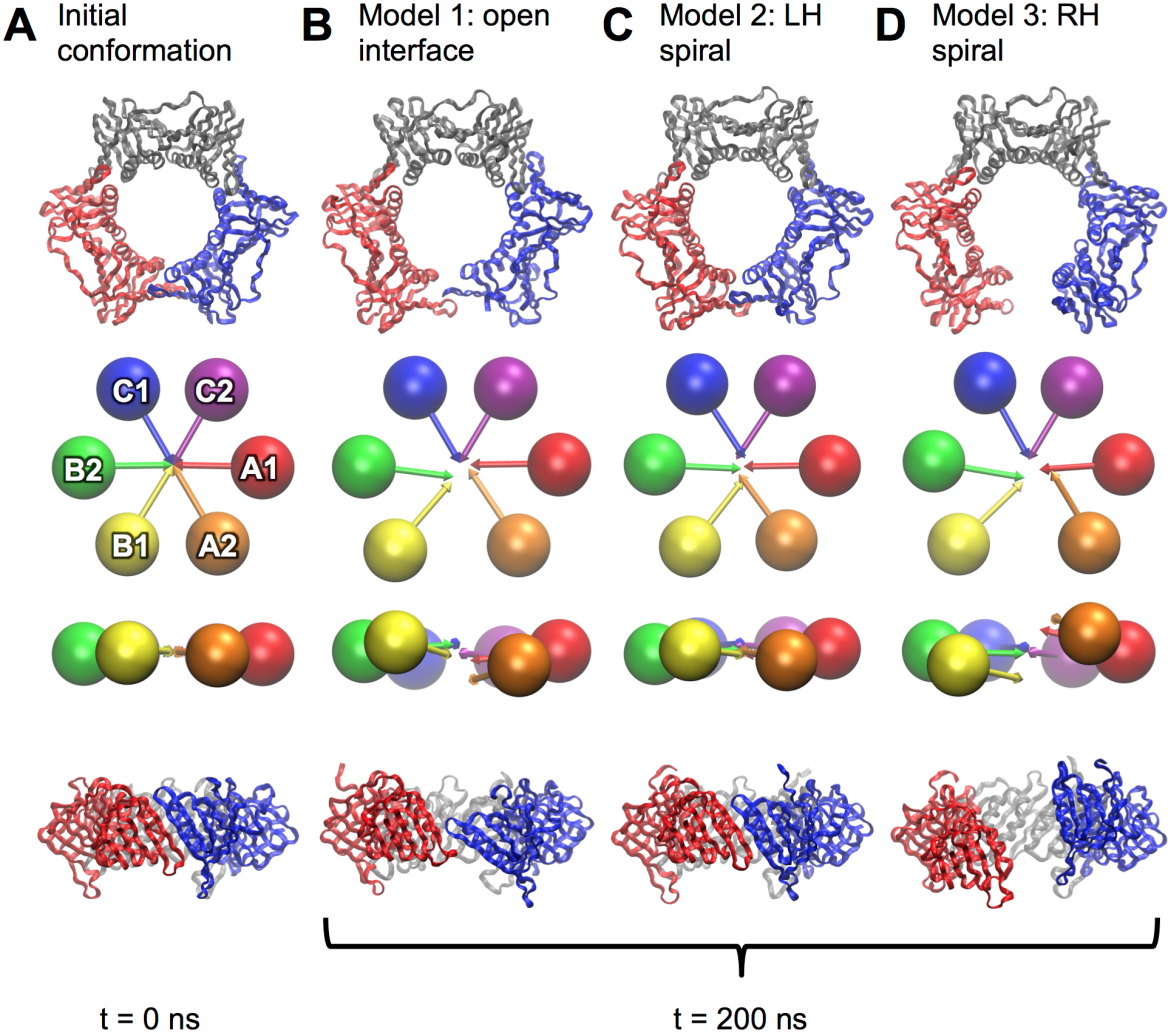
Structures of PCNA before and after ring-opening simulations. Initial conformation shown in (A) or after 200 ns of equilibration for models of type 1 (B), type 2 (C) and type 3 (D). All clamp domains are represented as ribbons and as “spheres and arrows” as described in methods. Pairs of orthogonal views are shown in all cases.

PCNA opened by steering the interface apart (model 1) formed a subtle right-handed spiral conformation (Fig 8B). Like β, the interface strands (βI_1_ in domain B1; βD_2_ in domain A2) unzip from the N-face upward during opening. With further equilibration, hydrophobic residues in the loop at the N-terminal end of strand βD_2_ contact βI_1_ in domain B1. When PCNA was opened as a left-handed spiral, there was a concerted breaking of the interface and rapid dissociation of domains A2 and B1. Equilibration of the open left-handed spiral form of PCNA led to a rapid flattening of the ring and closure of the interface (Fig 8C). The re-associated interface differed from the native structure in the relative translation of the βI_1_ and βD_2_ strands. When PCNA was opened as a right-handed spiral, the domain interfaces slid past each other. Surprisingly, the opened clamp did not close after further equilibration, but remained open, slowly drifting into a flat, open conformation with a slight left-handed spiral distortion (Fig 8D).

## Discussion

Equilibrium and steered molecular dynamics simulations on DNA-clamps suggest that while closed clamps are stable on the nanosecond timescale, clamps from different sources exhibit different behaviors and structural preferences in open conformations, and in incomplete sub-assemblies.

**Fig 9:**
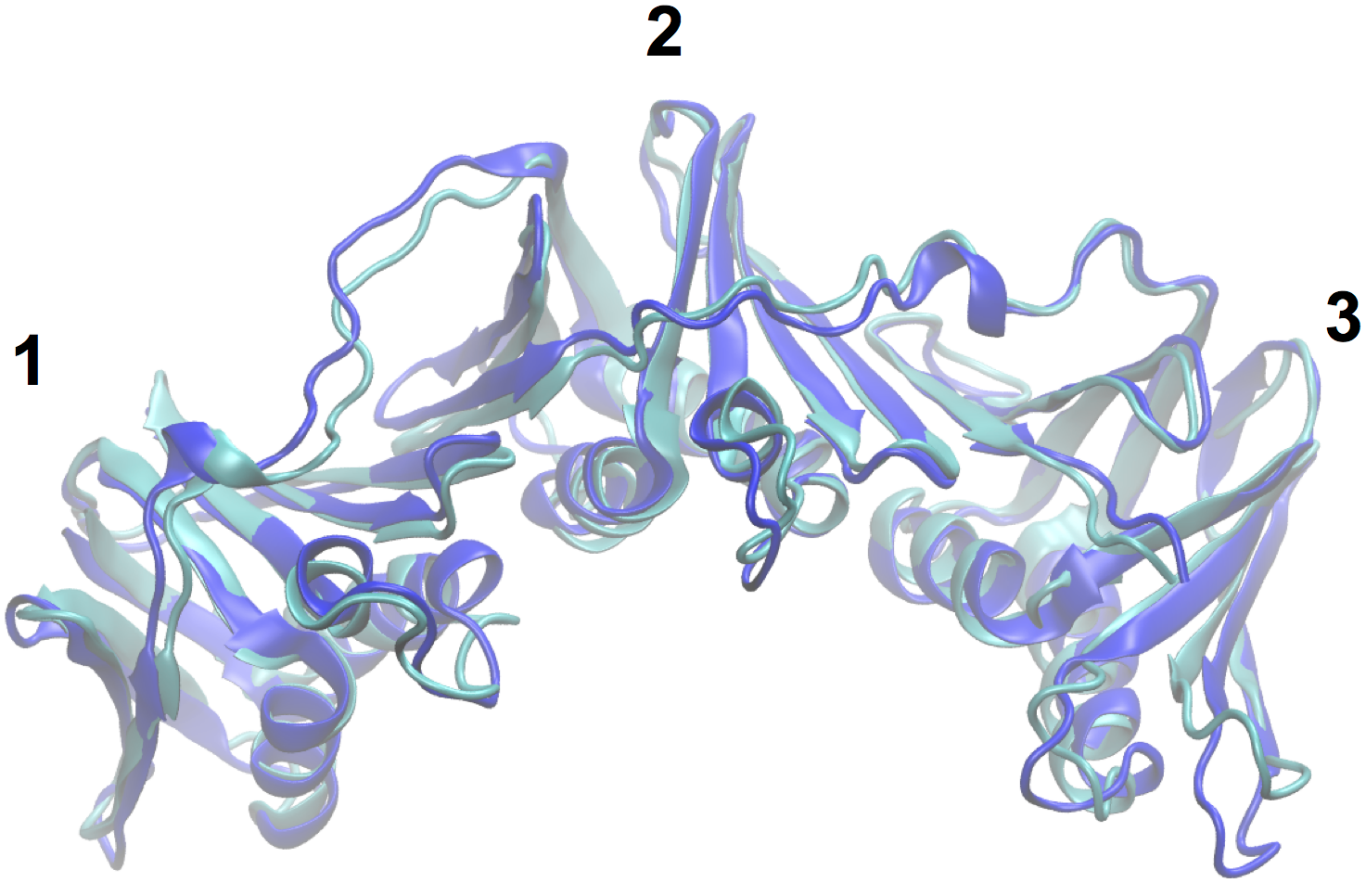
Comparison of monomeric β structures determined by crystallography and simulation. The monomeric (mutant) form of β from the crystal structure of the complex with clamp-loader subunit Pol III δ (PDB ID 1JQL) is shown in cyan. The monomer of β after 100 ns of equilibration is shown in blue. The clamp domains are labeled (1, 2 and 3).

The observation of greater flexibility of the monomeric β is in excellent agreement with hydrogen-deuterium exchange experiments showing the β monomer is more dynamic in solution in comparison to the dimer [21]. The simulated monomeric β showed a tendency to open up relative to the closed clamp. This opening was due to an increase in the interdomain (A1-A2-A3) angle of about 7°. A similar degree of opening (about 5°) was observed in the crystal structure of a monomeric (mutant) form of β in complex with clamp-loader subunit Pol III δ (PDB ID 1JQL) and simulation of the β monomer [42]. Fig 9 shows the monomeric forms of β from the crystal structure and simulation superimposed. These observations are matched by single-molecule polarization experiments on Cy3-labeled β in complex with its clamp loader [43], which revealed that domain 3 of the clamp rotated in-plane by approximately 8° during clamp closure. Therefore, the β monomer structures may be similar to those observed in the open β dimers found during clamp loading. Pol III δ in isolation is sufficient for opening of β dimers [44], suggesting that the β dimer represents a strained conformation and that the interaction with Pol III δ traps or induces conformations at the adjacent dimer interface allowing the ring to spring open when released [42]. Closed β rings are stable in solution, with a subunit dissociation *K*_d_ < 60 pM [24]. The calculations described here suggest that the β dimer is stable in the closed form and that isolated open clamps have a tendency to re-close. While the β monomer may prefer to adopt a shallower curve in isolation, the formation of energetically favorable hydrogen bonds and salt bridges across the inter-domain interface together with hydrophobic interactions appear to favor the closed conformation in dimeric β. In the equilibrium between closed and open states, binding of Pol III δ may stabilize the open conformation relative to closed. The strain induced by closing β monomers may help to tune the free energy difference between the closed and open states.

Simulations of the gp45 clamp indicate a clear preference for an open conformation in solution. Equilibration of monomers and dimers results in inter-domain rotations and conformations consistent with a right-handed spiral. Opening of flat, closed clamps also results in a right-handed spiral configuration, regardless of how the interfaces were opened. Intriguingly, a similar open conformation was observed in the crystal structure of gp45 with the gp44/gp62 clamp loader [20]. The simulations suggest that the open gp45 conformation is pre-configured to fit the binding surface of the gp44/62 clamp loader. The simulated structure of the gp45 clamp with two closed and one open interface is also in agreement with the trFRET experiments [26, 27]. In those experiments, the distances between a FRET donor (W92) and acceptor (7-diethylamino-3-(4’-maleimidylphenyl)-4-methylcoumarin covalently attached to C162) were determined to be 17 Å (closed interface) and 42 Å (open interface). These distances agree very well with equivalent distances in the simulated open clamp (closed interface 14 Å; open interface 36 Å) and the gp45-gp44/gp62 crystal structure (closed interface 13 Å; open interface 34Å). While the observation of closed and open interfaces in gp45 has been ascribed to a weak interface between protomers [45] the observations could be explained by the closed gp45 clamp being in a strained conformation relative to the open, with the clamp open at one interface representing a lower energy conformation. In the simulation of the closed gp45 clamp, the non-bonded interactions at the interfaces between protomers are sufficient to overcome the tension. On longer timescales inaccessible to current MD simulations, fluctuations would be expected to overcome these forces and produce spontaneous clamp opening. The question arises as to why all three gp45 inter-subunit interfaces are closed in all crystal structures of isolated clamps. The effects of precipitants used in crystallization may explain this: they compete with proteins for water molecules rendering protein–protein interactions (crystal contacts) more thermodynamically favorable. In the context of crystals of gp45 clamps, the third closed interface observed in gp45 crystal structures could therefore be regarded as an additional crystal contact. Packing effects in the crystal could also favor the closed conformation.

Evidence from small-angle neutron scattering (SANS) suggests that eukaryotic PCNA exist as a closed ring in solution [46]. Short (10 ns) simulations of PCNA dimers from yeast, human and the archaeon *Pyrococcus furiosus* with one subunit removed (i.e. dimers) showed oscillations between lateral openings and right-handed spirals [29]. The modeling presented here suggests that PCNA may open laterally, in right-handed or in left-handed spiral conformations. Our observation that PCNA is able to adopt both left- and right-handed spirals is in agreement with recent all-atom and coarse-grained simulations of yeast PCNA, suggesting that this clamp is not biased toward a right-handed conformation, that the clamp is flexible and the open clamp is “mechanically compliant in the clamp opening process” [30].

Cooperative monomer-to-trimer assembly is observed for both gp45 and PCNA clamps [26, 28]. Thus, dimers of gp45 and PCNA are expected to dissociate readily into monomers. Nevertheless, dissociation of dimers was not observed in the simulations described here. The timescale of the simulations (100 ns) appears to be too brief to observe this phenomenon.

It was once assumed that clamp loader complexes use the energy from ATP hydrolysis to open sliding clamps, but it is now recognized that ATP hydrolysis serves primarily to switch clamp loaders from high- to low-affinity DNA-binding modes [45]. Comparison of real time binding and opening reactions shows that the *E*. *coli* clamp loader complex first binds to and then opens the β clamp, and that ATP binding promotes this activity [47]. Binding of ATP to gp44/62 allows it to undergo a conformational change that permits interaction with the C-terminal face of an open gp45 clamp [48]. ATP binding to gp44/gp62 is sufficient for both binding to gp45 and assembly on DNA. The gp45 inter-subunit distance expands and contracts in response to ATP hydrolysis by gp44/62 [25]. Previously reported biophysical data [26, 27] and the simulations presented here suggest that the gp45 clamp is most relaxed in the open state, implying that work must be done by the gp44/gp62 clamp loader to close the clamp. However, ATP hydrolysis does not appear to be required for closure of gp45 as part of the complex with its clamp loader. Rather, ATP hydrolysis appears essential for the exit of gp44/62 from DNA after recruitment of the T4 DNA polymerase (gp43) to the gp45 clamp [25]. Similar to other clamp loaders, the binding of ATP to the yeast RFC promotes binding to PCNA, and ATP binding—but not hydrolysis—is required for opening of the PCNA ring [49]. Simulations of yeast PCNA opening in complex with RFC suggest that the clamp loader does not significantly destabilize the closed state of PCNA but instead selectively stabilizes the open conformation of the clamp [50]. This suggests that, as with Pol III δ binding to the β-clamp, an open form of PCNA is selectively stabilized by clamp loader binding.

Given the relative stability of closed β-clamp and PCNA rings, it may be hypothesized that the *E*. *coli* and eukaryotic RFC clamp loaders release their clamps, which then spontaneously close around primer–template junctions [51, 52]. Electrostatic interactions between the positively charged residues lining the inner surface of sliding clamps the negatively charged DNA backbone will favor contraction of open sliding clamps around DNA. FRET and other experimental data suggest that in the case of gp45, the ring remains partially open and that gp43 binds by inserting its C-terminus into the open subunit interface and docks onto the C-terminal face of gp45 [53]. This is inconsistent with the crystal structure of the gp45 homolog from RB69 in complex with a peptide derived from the RB69 DNA polymerase C-terminus, in which the clamp is closed and the polymerase peptide binds on the surface in a hydrophobic pocket [34]. If the closed structure is relevant to function, then the gp44/62 complex must actively close the gp45 clamp, rather than simply dissociate from it. Furthermore, electrostatic interactions between the basic residues in gp45 and the DNA backbone may favor the closed clamp conformation.
